# Transcription Profiling of Cultured *Acropora digitifera* Adult Cells Reveals the Existence of Ancestral Genome Regulatory Modules Underlying Pluripotency and Cell Differentiation in Cnidaria

**DOI:** 10.1093/gbe/evab008

**Published:** 2021-01-27

**Authors:** Alejandro Reyes-Bermudez, Michio Hidaka, Alexander Mikheyev

**Affiliations:** 1 Programa de Biologia, Universidad de la Amazonia, Florencia, Colombia; 2 Department of Chemistry, Biology, and Marine Science, University of the Ryukyus, Okinawa, Japan; 3 Ecology and Evolution Unit, Okinawa Institute of Science and Technology, Okinawa, Japan; 4 Research School of Biology, Division of Ecology and Evolution, Australian National University, Canberra, ACT, Australia

**Keywords:** coral cell culture, RNA-seq, coral stem cells, transcription regulation, cell differentiation

## Abstract

Due to their pluripotent nature and unlimited cell renewal, stem cells have been proposed as an ideal material for establishing long-term cnidarian cell cultures. However, the lack of unifying principles associated with “stemness” across the phylum complicates stem cells’ identification and isolation. Here, we for the first time report gene expression profiles for cultured coral cells, focusing on regulatory gene networks underlying pluripotency and differentiation. Cultures were initiated from *Acropora digitifera* tip fragments, the fastest growing tissue in *Acropora*. Overall, in vitro transcription resembled early larvae, overexpressing orthologs of premetazoan and *Hydra* stem cell markers, and transcripts with roles in cell division, migration, and differentiation. Our results suggest the presence of pluripotent cell types in cultures and indicate the existence of ancestral genome regulatory modules underlying pluripotency and cell differentiation in cnidaria. Cultured cells appear to be synthesizing protein, differentiating, and proliferating.

**Significance**

The inability to establish permanent cnidarian cell lines has directed attention to tissues with a high abundance of stem cells as an ideal material for establishing long-term cultures. Despite this, the lack of unifying principles associated with “stemness” across cnidaria complicates stem cells’ identification and isolation. Here, we report gene expression profiles for cultured coral cells. Our results revealed pluripotent cell types in cultures, identifying coral orthologs of stem cell markers that could be used for further isolation and characterization.

## Introduction

Cell–cell interactions are fundamental for body plan establishment and function as they integrate cell type–specific genome regulation during animal development (Peter and Davidson 2011). Coordination and assembly of cell-specific transcription profiles generate interconnected transcriptional networks that in cnidaria generate organismal complexity by deploying morphogenetic borders along an oral–aboral axis (Hayward et al. 2015). In cnidaria, tissue morphogenesis and homeostasis mechanisms are very diverse. The phylum exhibits complex life cycles and diverse developmental mechanisms, spanning planula (P), polyp, and medusa morphologies (Daly et al. 2007). Anthozoans, in particular, display complex polyp morphologies and less regenerative potential, requiring signaling from an organizer region to reconstruct their tissues (Hayward et al. 2015).

At the cellular level, although hydrozoans maintain body structure via the integration of three distinct stem cell lineages established during gastrulation—two epithelial and one interstitial (Hemmrich et al. 2012)—nonhydrozoans cnidarians present two epithelial stem cell lineages lacking the interstitial cell type (i-cells) (review in Gold and Jacobs 2013). Despite this, it is accepted that transdifferentiation of epithelial cells and dedifferentiation of “committed” cell types predates the evolution of cnidarian stem cell systems (Gold and Jacobs 2013). In animals, precursor cells in vivo maintain “stemness” and control differentiation via complex cell–cell interactions within a tissue grade microenvironment known as “stem-cell niche” (Fuchs et al. 2004; Gattazzo et al. 2014). Within the niche, differential deployment of core networks regulates cellular identity by promoting the expression of “pluripotency” genes while repressing developmental signaling pathways (Orkin and Hochedlinger 2011).

In metazoa, stem cells have been reported as early as porifera, with archeocytes and choanocytes identified as the oldest animal stem cell system (Funayama 2010). In cnidaria, undifferentiated stem cells have been reported in a variety of adult (Mydlarz et al. 2008) and larval (Martin and Chia 1982) tissues, with transcriptional profiles of precursor cell types characterized for *Hydra* (Siebert et al. 2019) and *Nematostella* (Sebé-Pedrós et al. 2018). Due to the high abundance of stem cells in adult stages, adult tissues have been suggested as an ideal material for the establishment of long-term cnidarian cultures (Rinkevich 2011). However, the lack of unifying principles associated with stemness across the phylum complicates identification and isolation of cnidarian stem cells for culture.

In cnidaria, attempts to establish permanent cnidarian cell lines have failed due to decreasing in vitro viability and proliferation (review in Rinkevich 2011). Despite this, short-term primary cultures have been used to understand the cellular mechanisms underlying fundamental cnidarian processes, such as symbiosis (Barnay-Verdier et al. 2013), calcification (Domart-Coulon et al. 2001; Mass et al. 2012), thermal stress (Nesa and Hidaka 2009), and regeneration (Schmid and Alder 1984). These studies reported inconsistent results regarding survival, proliferation, and viability, reflecting the lack of standardized protocols (reviewed by Rinkevich 2005, 2011). Nonetheless, as primary cell cultures are taken directly from in vivo tissues, they represent a powerful tool to study cellular and physiological processes unattainable using whole organisms.

This paper, reports for the first time gene expression profiles for cultured coral cells, focusing on regulatory gene networks underlying pluripotency and differentiation. We initiated primary cell cultures from *Acropora digitifera* tip fragments, the fastest growing tissue in *Acropora* corals (Lirman et al. 2014). *A. digitifera* has become an emerging model to study coral responses to environmental change (Shinzato et al. 2011) and due to its basal phylogenetic position in cnidaria (Bridge et al. 1992), the species is also an ideal model to study the evolution of animal developmental mechanisms.

Overall, after 4 weeks of culture, we conducted quantitative RNA-seq analysis and compared the in vitro transcriptome with a previously published *A. digitifera* developmental time series (Reyes-Bermudez et al. 2016). In vitro transcription closely resembled gene expression used in early larvae during the establishment of larval cellular phenotypes. Likewise, we identified upregulation in vitro of orthologs of premetazoan and *Hydra* stem cell markers (HM) and transcripts with roles in DNA replication, cell division, migration, and differentiation. Our results suggest the existence of 1) pluripotent cell types in cultures and 2) ancestral genome regulatory modules underlying pluripotency and cell differentiation in cnidaria.

## Results and Discussion

### Cultures Consist of Multicellular Aggregates Actively Dividing and Differentiating

After 4 weeks, cultures consisted of cellular aggregates formed by cells displaying a previously reported unique and nonspecific small round morphology (Reyes-Bermudez and Miller 2009). Cells did not attach to the substrate, and signs of CaCO_3_ precipitation were not observed. Contrasting with previous results (Lecointe et al. 2013), cultures did not show evidence of decreased proliferation. The fact that one round of subculturing was necessary for 2 weeks after initiation, suggests that cells were actively dividing. Although zooxanthellae were present abundantly at initiation, after 4 weeks, chlorophyll fluorescence was absent. Our results support the idea that the capacity to maintain or reconstruct cell signaling between epitheliums in vitro is critical for the establishment of “long-term” successful primary cultures (reviewed by Rinkevich 2005, 2011).

Moreover, upregulation in vitro of genes with roles in protein synthesis, proliferation, and differentiation indicate that, at least after 4 weeks, a subset of cells was proliferating and differentiating in cultures (table 1). The fact that cultures were initiated from branch tips, the fastest growing tissue in *Acropora* corals (Lirman et al. 2014), suggest that undifferentiated cell populations in founder tissues might be responsible for in vitro enrichment of corals cells that is a crucial factor for culture viability (reviewed by Rinkevich 2005, 2011). Reduced proliferation in primary cnidarian cultures has been linked to a decrease in the proportion of animal cells as result of culture contamination (Frank et al. 1994).

**Table 1:** Gene Ontology (GO) Enrichment Summary in DEGs Only Upregulated in Cells

GO ID	Node Size	Sample Match	*P* Adj	Term	Ontology
GO:0000302	990	421	2.02E−09	Response to reactive oxygen species	BP
GO:0006415	152	92	2.20E−09	Translational termination	BP
GO:0006414	369	173	6.60E−06	Translational elongation	BP
GO:0042755	258	125	0.000134999	Eating behavior	BP
GO:0043462	293	131	0.018485676	Regulation of ATPase activity	BP
GO:0000302	990	421	2.02E−09	Response to reactive oxygen species	BP
GO:0006415	152	92	2.20E−09	Translational termination	BP
GO:0006414	369	173	6.60E−06	Translational elongation	BP
GO:0042755	258	125	0.000134999	Eating behavior	BP
GO:0043462	293	131	0.018485676	Regulation of ATPase activity	BP
GO:0009725	2914	1,050	0.000676117	Response to hormone stimulus	BP
GO:0030865	350	181	4.75E−11	Cortical cytoskeleton organization	BP
GO:0003012	826	364	2.70E−10	Muscle system process	BP
GO:0050905	485	202	0.020936066	Neuromuscular process	BP
GO:0002026	161	86	8.57E−05	Regulation of the force of heart contraction	BP
GO:0060327	118	69	1.68E−05	Cytoplasmic actin-based contraction involved in cell motility	BP
GO:0002027	337	158	3.54E−05	Regulation of heart rate	BP
GO:0000916	184	95	0.000144077	Actomyosin contractile ring contraction	BP
GO:0007109	148	82	1.95E−05	Cytokinesis, completion of separation	BP
GO:0032060	158	81	0.002337013	Bleb assembly	BP
GO:0048739	153	80	0.000935569	Cardiac muscle fiber development	BP
GO:0035277	134	72	0.000968262	Spiracle morphogenesis, open tracheal system	BP
GO:0007427	218	105	0.00265387	Epithelial cell migration, open tracheal system	BP
GO:0046664	128	70	0.000572738	Dorsal closure, amnioserosa morphology change	BP
GO:0007395	123	69	0.000180261	Dorsal closure, spreading of leading edge cells	BP
GO:0007496	115	66	0.000106495	Anterior midgut development	BP
GO:0021549	341	169	4.16E−08	Cerebellum development	BP
GO:0030224	166	95	9.18E−08	Monocyte differentiation	BP
GO:0002119	798	341	2.08E−07	Nematode larval development	BP
GO:0030220	127	72	4.86E−05	Platelet formation	BP
GO:0007527	125	71	5.55E−05	Adult somatic muscle development	BP
GO:0045200	141	77	0.000140176	Establishment of neuroblast polarity	BP
GO:0048147	35	25	0.014716604	Negative regulation of fibroblast proliferation	BP
GO:0001767	116	66	0.000170584	Establishment of lymphocyte polarity	BP
GO:0006930	130	74	2.35E−05	Substrate-dependent cell migration, cell extension	BP
GO:0033057	527	224	0.000940241	Multicellular organismal reproductive behavior	BP
GO:0051015	403	203	1.23E−11	Actin filament binding	MF
GO:0008307	210	109	2.95E−06	Structural constituent of muscle	MF
GO:0019829	394	179	2.23E−05	Cation-transporting ATPase activity	MF
GO:0031762	119	68	2.73E−05	Follicle-stimulating hormone receptor binding	MF
GO:0008013	155	80	0.000637174	Beta–catenin binding	MF
GO:0043531	316	143	0.000915446	ADP binding	MF
GO:0005167	47	30	0.017776487	Neurotrophin TRK receptor binding	MF
GO:0005516	616	286	2.94E−11	Calmodulin binding	MF
GO:0051020	651	279	3.30E−06	GTPase binding	MF
GO:0005083	586	245	0.000425907	Small GTPase regulator activity	MF
GO:0019901	1,428	583	1.59E−10	Protein kinase binding	MF
GO:0004725	363	157	0.007252685	Protein tyrosine phosphatase activity	MF

Note.—Node size = total number of GO terms in node. Sample match = number of transcripts with GO terms associated to specific nodes.

### Cultured Cells Express More Genes in Common with Early Larvae Than with Adult, P, or Embryonic Stages

**Fig. 1.**
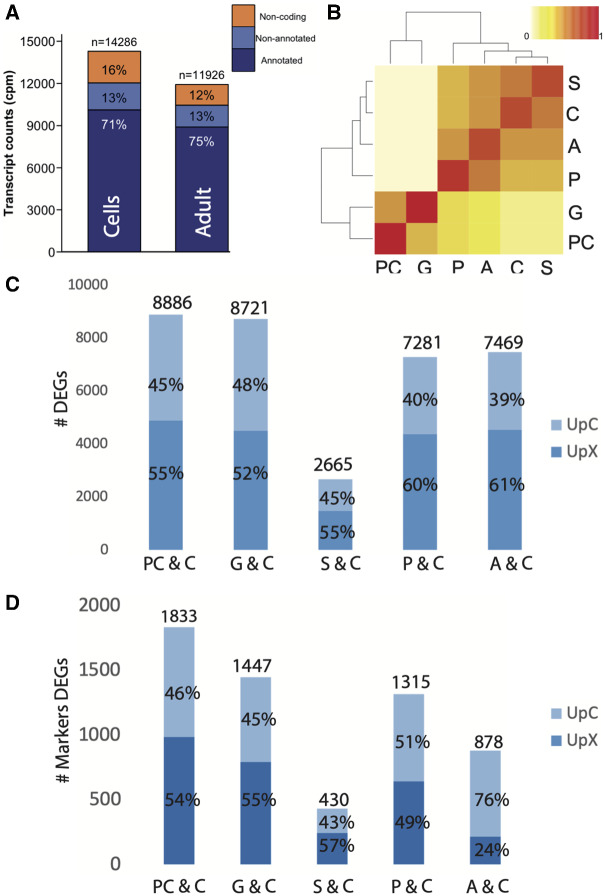
In vitro transcriptome characterization. Cells’ specific transcription profile resulted in 14,286 transcripts compared with 11,926 reported for adult tissue (*A*). Transcriptome comparison between cultured cells and *Acropora digitifera* developmental stages showed that although cultures were initiated from adult tissues, their expression profile was closer to early larvae (S) than to any other in vivo stage (*B*). Differential gene expression analysis revealed a lower number of DEGs in the SvsC comparison (*C*). Only a fraction of DEGs were identified as orthologs of HM. The lowest percentage was observed in AvsC and the highest in PCvsC (*D*). Blastula, PC; gastrula, G; early larvae, S; planula, P; adult, A; upregulated in C, UpC; upregulated in vivo, UpX.

In vitro transcription profile resulted in 14,286 transcripts compared with 11,926 observed in adult tissue (fig. 1*A*). Transcriptome comparison between cultured cells (C) and data on previously published *A. digitifera* stages (Reyes-Bermudez et al. 2016), showed that in vitro gene expression is closer to early larvae (sphere[S]) than to adult polyps (adult [A]) (fig. 1*B*). These results suggest that the abolishment of morphogenetic borders following disassociation of coral polyps resulted in in vitro overexpression of transcriptional networks that resemble those used by S (Reyes-Bermudez et al. 2016). Differential expression analysis supported the observation, showing that C cells expressed more genes in common with S than with other stages (fig. 1*C*). Clustering of embryonic transcriptomes (blastula—prawnchip-like blastula [PC] and gastrula—G) as a distinct group that differs from the remaining stages, indicates that C cells are most likely cellular lineages originated after gastrulation (fig. 1*B*).

Moreover, enrichment in the subset of differentially expressed genes (DEGs) upregulated in vitro with molecules involved in diverse morphogenetic and differentiation processes (table 1) indicates uncoordinated overexpression of genome regulatory programs following the loss of morphogenetic borders (Beloussov 2015). Results revealed a transcriptionally heterogenous C cell population that differed transcriptionally from the in vivo system. Significant transcriptional changes that reflect the emergence of more active and proliferative subgroups have been reported in cell cultures over time (Januszyk et al. 2015).

### Cells Expressed Orthologs of Premetazoan and HM

**Fig. 2.**
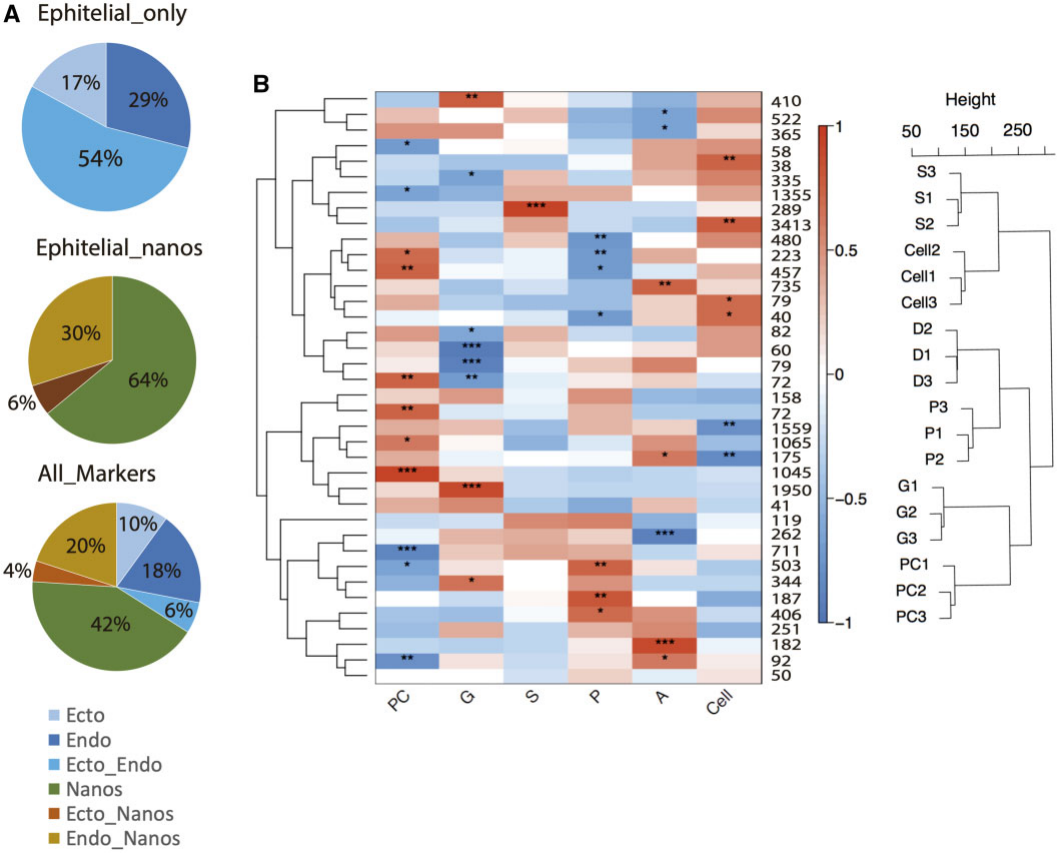
Orthologs of HM and coexpression networks. The HM fraction expressed in vitro was enriched by both endodermal and i-cell HM (*A*). Transcripts (18,264) were assigned to 38 different gene modules that ranged from 38 to 3,413 transcripts and grouped in two main coexpression clusters (C1 and C2). Eigengenes were calculated for each module and although we were able to identify discrete gene expression patterns, in most cases. significant module–trait correlations were observed in a stage-specific fashion. *P value ≤0.05, **P value ≤0.01, ***P value ≤0.01. Blastula, PC; gastrula, G; early larvae, S; planula, P; adult, A; cultured cells, C.

Consistent with 500 Myr of independent cnidarian evolution (Steele et al. 2011), only a small fraction of DEGs were identified as orthologs of HM. We observed over expression of a higher number of HM in Blastula (PC) (fig. 1*D*), which is consistent with the predominant pluripotent cellular phenotypes present at the stage. Interestingly, *Acropora* HM orthologs upregulated in vitro were enriched with markers overexpressed by *Hydra*’s i-cells (nanos) (fig. 2*A*), which, in a strict sense, is the only true characterized cnidarian stem cell population (Frank et al. 2009). These results may not indicate the presence in coral tissues of stem cell populations homologous to *Hydra*’s i-cells but most likely indicate the utilization of conserved molecules underlying pluripotency and cell differentiation in *Acropora.*

Similarly, we observed enrichment in cultures of orthologs of endodermal markers such as Brachyury (Yasuoka et al. 2016) and Hedgehog (Matus et al. 2008) (supplementary material S1–S4, Supplementary Material online). Whether this reflects endodermal enrichment in vitro or utilization of conserved transcriptional networks by different lineages, is not clear. For example, although i-cells are thought to be a “recent” cnidarian innovation, their transcriptome is phylogenetically older than those of *Hydra’*s epithelial lineages (Hemmrich et al. 2012), suggesting recruitment of ancestral regulatory networks during *Hydra’*s i-cell evolution. Our results support this idea and suggest that regulatory networks associated with maintenance of pluripotency and differentiation are not fixed entities linked to specific cell types, but dynamic modules recruited and modified multiple times by natural selection during cnidarian diversification.

Likewise, identification of premetazoan and metazoan stem cell markers in the subset of DEGs exclusively upregulated in vitro (table 2) is consistent with the idea that animal stem cell systems were built upon ancestral regulatory gene networks present in the last common metazoan ancestor (Alié et al. 2015). Upregulation in vitro of *Acropora* orthologs with roles in cell cycle, replication, chromosome maintenance, stress response, DNA repair, as well as diverse transcripts coding RNA-binding proteins, such as a Musashi-1 ortholog (table 2), imply that components of regulatory gene networks associated to stemness are being expressed in cultures.

**Table 2:** DEGs with Putative Roles in Stem Cell Homeostasis

ID	Annotation	KEGG	Marker	Function
**Ancestral genes**				
adi_v1.04467	Nuclear factor NF-kappa-B p105 subunit—Metazoa	K02580	Nanos_3529	Cell growth and differentiation
adi_v1.12580	Heterogeneous nuclear ribonucleoprotein K—Metazoa	K12886	Nanos_2219	Pre-mRNA splicing
adi_v1.12078	ATP-dependent RNA helicase DDX56/DBP9 - Eukariota	K14810	Nanos_1725	RNA-Helicases
adi_v1.19896	DNA polymerase alpha subunit A—Eukariota	K02320	Nanos_3477	DNA replication
adi_v1.00008	Replication factor C subunit 3/5—Eukariota	K10756	Nanos_2523	DNA repair
adi_v1.17163	Flap endonuclease-1—Pre-Eukariota	K04799	Nanos_Ecto_497	DNA repair
**Replication**				
adi_v1.13235	Replication factor C subunit 2/4	K10755	Nanos_2940	Replication
adi_v1.00008	Replication factor C subunit 3/5	K10756	Nanos_2523	Replication
adi_v1.19896	DNA polymerase alpha subunit A (EC:2.7.7.7)	K02320	Nanos_3477	Replication
adi_v1.02983	DNA polymerase sigma (EC:2.7.7.7)	K03514	Nanos_74	Replication
adi_v1.10030	DNA polymerase zeta (EC:2.7.7.7)	K02350	Nanos_4979	Replication
adi_v1.24240	ATP-binding protein involved in chromosome partitioning	K03593	Nanos_1409	Replication
adi_v1.13671	DNA polymerase zeta (EC:2.7.7.7)	K02350	Endo_Nanos_16	Replication
adi_v1.12265	DNA topoisomerase VI subunit B (EC:5.99.1.3)	K03167	Endo_Nanos_1274	Replication
adi_v1.19036	DNA polymerase sigma (EC:2.7.7.7)	K03514	Endo_Nanos_861	Replication
**Cell cycle**				
adi_v1.05785	Cell cycle arrest protein BUB3	K02180	Nanos_5104	Cell cycle
adi_v1.04546	Cell cycle checkpoint protein	K06662	Nanos_2749	Cell cycle
XLOC_000501	Cell division cycle 20-like protein 1, cofactor of APC complex	K03364	Nanos_7115	Cell cycle
XLOC_019254	Cell division cycle 20-like protein 1, cofactor of APC complex	K03364	Nanos_1396	Cell cycle
adi_v1.03932	Cell division cycle 20-like protein 1, cofactor of APC complex	K03364	Nanos_10963	Cell cycle
adi_v1.06900	G1-/S-specific cyclin PLC1	K06656	Nanos_1058	Cell cycle
adi_v1.13930	Centromere protein B	K11496	Nanos_2602	Cell cycle
adi_v1.09992	Cell division protein ZapA	K09888	Endo_Nanos_2012	Cell cycle
adi_v1.24600	Signal-induced proliferation-associated gene 1	K08013	Endo_Nanos_1253	Cell cycle
adi_v1.24600	Signal-induced proliferation-associated gene 1	K08013	Endo_Nanos_1253	Cell cycle
XLOC_020915	Cell division cycle 20-like protein 1, cofactor of APC complex	K03364	Ecto_3004	Cell cycle
**Helicases**				
adi_v1.01424	Chromodomain–helicase–DNA-binding protein 7 (EC:3.6.4.12)	K14437	Nanos_422	Chromatin remodeling
adi_v1.01424	Chromodomain–helicase–DNA-binding protein 7 (EC:3.6.4.12)	K14437	Nanos_422	Chromatin remodeling
adi_v1.13340	RNAi-mediated heterochromatin assembly 1 (EC:3.6.4.13)	K11701	Endo_Ecto_43	Chromatin remodeling
adi_v1.23884	ATP-dependent RNA helicase DHX8/PRP22 (EC:3.6.4.13)	K12818	Nanos_1508	Splicing/transcription
adi_v1.08670	ATP-dependent RNA helicase DHX15/PRP43 (EC:3.6.4.13)	K12820	Nanos_806	Splicing/transcription
adi_v1.23237	ATP-dependent RNA helicase DDX1 (EC:3.6.4.13)	K13177	Nanos_1406	Splicing/transcription
adi_v1.12078	ATP-dependent RNA helicase DDX56/DBP9 (EC:3.6.4.13)	K14810	Nanos_1725	Ribosome biogenesis
**Chromosome maintenance**				
adi_v1.24240	ATP-binding protein involved in chromosome partitioning	K03593	Nanos_1409	Chromosome maintenance
adi_v1.17702	Structural maintenance of chromosome 1	K06636	Nanos_Ecto_213	Chromosome maintenance
adi_v1.15153	Structural maintenance of chromosome 4	K06675	Nanos_Ecto_50	Chromosome maintenance
adi_v1.01018	Structural maintenance of chromosome 1	K06636	Nanos_653	Chromosome maintenance
adi_v1.14806	Structural maintenance of chromosome 4	K06675	Nanos_3734	Chromosome maintenance
adi_v1.11026	Structural maintenance of chromosome 4	K06675	Nanos_6620	Chromosome maintenance
adi_v1.04706	Chromosome segregation protein	K03529	Endo_Nanos_897	Chromosome maintenance
adi_v1.01300	Chromosome transmission fidelity protein 1 (EC:3.6.4.13)	K11273	Ecto_1702	Chromosome maintenance
adi_v1.21696	Chromosome segregation protein	K03529	Endo_Ecto_309	Chromosome maintenance
**DNA repair/stress response**				
adi_v1.23838	Three prime repair exonuclease 2 (EC:3.1.11.2)	K10791	Nanos_3244	DNA repair
adi_v1.03868	DNA damage-inducible protein 1	K11885	Nanos_1310	DNA repair
adi_v1.02191	DNA excision repair protein ERCC-2 (EC:3.6.4.12)	K10844	Nanos_1749	DNA repair
adi_v1.22737	DNA excision repair protein ERCC-3 (EC:3.6.4.12)	K10843	Nanos_3028	DNA repair
adi_v1.22267	DNA excision repair protein ERCC-4 (EC:3.1.-.-)	K10848	Nanos_1886	DNA repair
adi_v1.11724	DNA excision repair protein ERCC-8	K10570	Nanos_5126	DNA repair
adi_v1.03203	DNA excision repair protein ERCC-8	K10570	Nanos_6011	DNA repair
adi_v1.11542	DNA repair protein RAD16	K15083	Nanos_9086	DNA repair
adi_v1.06342	DNA repair protein RAD50 (EC:3.6.-.-)	K10866	Nanos_2601	DNA repair
adi_v1.19161	DnaJ homolog subfamily A member 5	K09506	Nanos_1092	Stress response
XLOC_001068	DnaJ homolog subfamily B member 9	K09515	Nanos_4628	Stress response
adi_v1.04788	Heat shock 70 kDa protein 1/8	K03283	Nanos_2410	Stress response
adi_v1.02262	Heat shock 70 kDa protein 1/8	K03283	Nanos_2410	Stress response
adi_v1.04284	Stress-induced-phosphoprotein 1	K09553	Nanos_2665	Stress response
**RNA-binding proteins**				
adi_v1.16723	Multiple RNA-binding domain-containing protein 1	K14787	Nanos_527	RNA-binding
adi_v1.13400	oo18 RNA-binding protein	K02602	Nanos_1849	RNA-binding
adi_v1.10220	RNA-binding protein 15	K13190	Nanos_1931	RNA-binding
adi_v1.03795	RNA-binding protein 39	K13091	Nanos_357	RNA-binding
adi_v1.05305	RNA-binding protein Musashi	K14411	Nanos_2716	RNA-binding
adi_v1.03308	RNA-binding protein 26	K13192	Endo_7052	RNA-binding
adi_v1.15031	U1 small nuclear ribonucleoprotein A	K11091	Nanos_334	RNA-binding
adi_v1.00705	U3 small nucleolar ribonucleoprotein protein IMP4	K14561	Nanos_985	RNA-binding
adi_v1.06360	U3 small nucleolar RNA-associated protein 20	K14772	Nanos_283	RNA-binding
adi_v1.19916	U3 small nucleolar RNA-associated protein 21	K14554	Nanos_2230	RNA-binding
adi_v1.13965	U3 small nucleolar RNA-associated protein 24	K14566	Nanos_1277	RNA-binding
adi_v1.12414	U3 small nucleolar RNA-associated protein 5	K14546	Nanos_200	RNA-binding
adi_v1.11136	U3 small nucleolar RNA-associated protein 6	K14557	Nanos_1283	RNA-binding
adi_v1.07136	U3 small nucleolar RNA-associated protein 6	K14557	Nanos_1283	RNA-binding
XLOC_015243	U3 small nucleolar RNA-associated protein 7	K14768	Nanos_1007	RNA-binding
adi_v1.17859	Heterogeneous nuclear ribonucleoprotein K	K12886	Nanos_2219	RNA-binding
adi_v1.09619	Heterogeneous nuclear ribonucleoprotein M	K12887	Nanos_141	RNA-binding
adi_v1.23861	U4/U6 small nuclear ribonucleoprotein SNU13	K12845	Nanos_Ecto_54	RNA-binding
adi_v1.21303	RNA-binding protein 15	K13190	Endo_Nanos_332	RNA-binding
adi_v1.12580	RNA-binding protein 5/10	K13094	Endo_Nanos_738	RNA-binding
adi_v1.07694	Heterogeneous nuclear ribonucleoprotein L	K13159	Endo_Nanos_24	RNA-binding
adi_v1.21839	Small nuclear ribonucleoprotein B and B'	K11086	Endo_1503	RNA-binding
**Ribosome biogenesis**				
adi_v1.12527	rRNA biogenesis protein RRP5	K14792	Nanos_669	Ribosome biogenesis
adi_v1.10921	rRNA biogenesis protein RRP5	K14792	Nanos_669	Ribosome biogenesis
adi_v1.20333	Regulator of ribosome biosynthesis	K14852	Nanos_751	Ribosome biogenesis
adi_v1.09262	Ribosome assembly protein 4	K14855	Nanos_1608	Ribosome biogenesis
adi_v1.05788	Ribosome biogenesis protein BMS1	K14569	Nanos_481	Ribosome biogenesis
adi_v1.01894	Ribosome biogenesis protein MAK21	K14832	Nanos_2059	Ribosome biogenesis
adi_v1.05433	Ribosome biogenesis protein NSA2	K14842	Nanos_425	Ribosome biogenesis
XLOC_014614	Ribosome production factor 1	K14846	Nanos_1610	Ribosome biogenesis
adi_v1.04696	Ribosome biogenesis GTPase A	K14540	Endo_Nanos_387	Ribosome biogenesis
adi_v1.02489	Ribosome biogenesis protein MAK21	K14832	Endo_Nanos_4397	Ribosome biogenesis

### Coexpression Modules Reveal Distinct G-/P-Specific Genome Regulatory Programs

Network analysis assembled DEGs in 38 modules within two main coexpression groups, consisting of distinct and diverse stage-specific coexpression clusters (fig. 2*B*). Coexpression units usually reflect common functionality and regulation (review in Peter and Davidson 2011). Interestingly, most in vitro upregulated DEG’s were coexpressed in PC, S, and A but were significantly downregulated in G and P stages (fig. 2*B*). Differential usage of enhancers between G and P stages have been reported for *Nematostella* (Schwaiger et al. 2014), suggesting the existence of distinct G-/P-specific genome regulatory programs in cnidaria. More research is necessary to test this idea as transcriptional networks underlying early morphogenetic transitions in metazoans are variable and, in some cases, taxa-specific (Erwin and Davidson 2009; Davidson 2010).

Finally, transcriptome comparisons using coexpression networks and transcript composition showed slightly different results. Although the topology built using complete transcriptomes clustered S and C as a sister group to A, leaving P as the most dissimilar stage and PC and G as a separate group, the topology based on coexpression data, resolved P and A as a sister group to PC and G (fig. 1*D*). In both cases, the similarity between C and S was clear, indicating the usage in the two stages of shared genome regulatory programs based on similar transcript composition. On the other hand, differences between topologies reveal that in vivo complexity is strongly dependent on network interactions and supports the idea that body plan morphogenesis and evolution is a “system-level problem” that cannot be understood by looking at developmental conserved genes in isolation (Peter and Davidson 2011).

## Conclusion

Our study demonstrated that primary coral cultures are valuable tools for studying genome regulatory programs and revealed the existence of ancestral genome regulatory modules underlying pluripotency and cell differentiation in cnidaria. Caution must be taken to interpret in vitro experiments as C populations are heterogeneous cell types that drastically differ transcriptionally from the in vivo system.

## Materials and Methods

### Collection of Samples and Tissue Culture

Tip fragments (∼3 cm) from six different colonies were kept in 50 ml falcon tubes containing 0.2 μm filtered seawater with antibiotics (FSWA) (1% Pen/Strep/L-Glu-solution, Sigma–Aldrich and 0.1% Fungizone, Invitrogen) prior the initiation of cultures. Samples were washed 3× with FSWA and then incubated at 32 °C for 4 h to induce bleaching (Desalvo et al. 2010). Fragments were washed (3×) with FSWA and further incubated (2 h/gentle shaking) in calcium-free FSWA (Marshall and Clode 2004). Following tissue dissociation, naked skeletons were removed, and detached tissue centrifuged (1,500 rpm/10 min) and resuspended in 5 ml of FSWA. Cell pellets were gently washed 3× with 5 ml of cell culture media (30% DMEM Gibco, 10% FBS Gibco, 1% Pen/Strep-solution, 0.1% Fungizone, 1% Glutamax, Gibco, 25 mM HEPES pH 8.0, and 55% 0.2 μm filtered seawater) and resuspended in 5 ml of fresh media. Founding cultures were kept individually in 6-well cultured plates and incubated at 23 °C in the dark for 48 h. After that, 1 ml of the original cultures were used to inoculate 4 ml of fresh media in 6-well cultured plates and returned to incubation conditions. Cultures were monitored daily on a standard inverted light microscope fitted with a color digital camera. Media was changed when cultures reached 60% confluence. After 4 weeks, cells from three wells were harvested for RNA extractions.

### Sequencing and Data Analysis

Library preparation and data analysis were conducted as reported in Reyes-Bermudez et al. (2016). To identify *Acropora* HM, we download the T-CDS data set from *Hydra vulgaris* strain AEP from http://www.compagen.org/datasets.html. Orthologs were determined using OrthoMCL v.1.4 with a BLASTp *E* value cut-off of 1e^−5^, a minimum coverage of 70% and an inflation index of 1.5 (Li et al. 2003).

## Supplementary Material

Supplementary data are available at *Genome Biology and Evolution* online.

## Supplementary Material

evab008_Supplementary_Data
